# Safety and tolerability of combination antimalarial therapies for uncomplicated falciparum malaria in Ugandan children

**DOI:** 10.1186/1475-2875-7-106

**Published:** 2008-06-11

**Authors:** Catherine Maiteki-Sebuguzi, Prasanna Jagannathan, Vincent M Yau, Tamara D Clark, Denise Njama-Meya, Bridget Nzarubara, Ambrose O Talisuna, Moses R Kamya, Philip J Rosenthal, Grant Dorsey, Sarah G Staedke

**Affiliations:** 1 Department of Medicine, Makerere University, Kampala, Uganda; 2 Department of Medicine, University of California, San Francisco, California, USA; 3 Department of Epidemiology and Statistics, University of California, Berkeley, California, USA; 4 Epidemiological Surveillance Division, Ministry of Health, Kampala, Uganda; 5 Department of Infectious and Tropical Diseases, London School of Hygiene & Tropical Medicine, Keppel Street, London, WC1E 7HT, UK

## Abstract

**Background:**

Combination antimalarial therapy is recommended for the treatment of uncomplicated *falciparum *malaria in Africa; however, some concerns about the safety and tolerability of new regimens remain. This study compared the safety and tolerability of three combination antimalarial regimens in a cohort of Ugandan children.

**Methods:**

A longitudinal, single-blind, randomized clinical trial of children was conducted between November 2004 and May 2007 in Kampala, Uganda. Upon diagnosis of the first episode of uncomplicated malaria, participants were randomized to treatment with amodiaquine + sulphadoxine-pyrimethamine (AQ+SP), artesunate + amodiaquine (AS+AQ), or artemether-lumefantrine (AL). Once randomized, participants received the same regimen for all subsequent episodes of uncomplicated malaria. Participants were actively monitored for adverse events for the first 14 days after each treatment, and then passively followed until their next study medication treatment, or withdrawal from study. Outcome measures included the risk of adverse events at 14 and 42 days after treatment.

**Results:**

Of 601 enrolled children, 382 were diagnosed with at least one episode of uncomplicated malaria and were treated with study medications. The median age at treatment was 6.3 years (range 1.1 – 12.3 years). At 14 days of follow-up, AQ+SP treatment was associated with a higher risk of anorexia, weakness, and subjective fever than treatment with AL, and a higher risk of weakness, and subjective fever than treatment with AS+AQ. Treatment with AL was associated with a higher risk of elevated temperature. Repeated episodes of neutropaenia associated with AS+AQ were detected in one participant. Considering only children less than five years, those who received AQ+SP were at higher risk of developing moderate or severe anorexia and weakness than those treated with AL (anorexia: RR 3.82, 95% CI 1.59 – 9.17; weakness: RR 5.40, 95% CI 1.86 – 15.7), or AS+AQ (anorexia: RR 2.10, 95% CI 1.04 – 4.23; weakness: RR 2.26, 95% CI 1.01 – 5.05). Extending the analysis to 42 days of follow-up had little impact on the findings.

**Conclusion:**

This study confirms the safety and tolerability of AS+AQ and AL in Ugandan children, and suggests that AQ+SP is safe, but less well-tolerated, particularly in younger children. As newer antimalarial regimens are deployed, collecting data on their safety and tolerability will be essential.

**Trial registration:**

Current Controlled Trials Identifier ISRCTN37517549

## Background

Combination antimalarial therapy is now advocated for treatment of uncomplicated falciparum malaria in Africa [1,2]. Of the five regimens currently recommended by the World Health Organization (WHO) for treatment of falciparum malaria, two artemisinin-based combination therapies (ACTs), artesunate + amodiaquine (AS+AQ) and artemether-lumefantrine (AL), have been adopted by most countries in sub-Saharan Africa [3]. A non-ACT regimen, amodiaquine + sulphadoxine-pyrimethamine (AQ+SP), is recommended as an alternative to ACT in areas where both drugs remain effective. This regimen benefits from its lower cost, and may be a good option for parts of West Africa [4-6].

Although combination therapy is highly effective, some concerns about the safety and tolerability of these regimens remain [7]. Serious toxicity has been associated with SP and AQ, particularly when used for long-term chemoprophylaxis, including severe cutaneous reactions with SP, and neutropaenia and hepatotoxicity with AQ [8-10]. However, both drugs appear to be much safer when used in short-term treatment regimens [11,12]. Artemisinin derivatives are generally safe and well-tolerated, but there are concerns about the potential for neurotoxicity and reproductive toxicity [13-15]. Lumefantrine, although closely related to halofantrine, does not appear to be cardiotoxic at therapeutic doses [16], and the AL combination appears to have a favourable safety profile [17,18].

Recent studies of the safety of newer combination regimens have been reassuring, identifying no serious concerns, but to date, assessments of drug safety and tolerability have been limited [19-22]. Most antimalarial safety data have been gathered in clinical trials evaluating treatment of single episodes of malaria. However, in practice, African children are treated for malaria repeatedly, raising concern for toxicity resulting from repeated short-term exposures [23]. The relatively small sample size of clinical trials may also limit the ability to detect uncommon events [24].

A longitudinal, single-blind, randomized controlled trial assessing the efficacy and safety of AQ+SP, AS+AQ and AL for all episodes of uncomplicated malaria was begun in November 2004 in a cohort of children in Kampala, Uganda [25]. An analysis of the efficacy data collected through June 2006 indicated that the 28-day risks of recurrent malaria differed significantly between the regimens (26%, 17%, and 7% for the AQ+SP, AS+AQ, and AL treatment groups, respectively) [25]. Here, detailed data on the safety and tolerability of these therapies, in the context of repeated treatments, is presented, with follow-up extended through May 2007.

## Methods

### Study site and recruitment

The study was conducted between November 2004 and May 2007 in the Mulago III parish of Kampala. Mulago III parish is primarily residential with a high population density, typical of an urban slum. The parish is located near the study clinic at Mulago Hospital, the main tertiary referral hospital for Uganda.

A census project was conducted prior to the onset of the study to generate a sampling frame of households with appropriately aged children for recruitment and to gather basic demographic information about the target population [26]. Experienced home visitors approached households for recruitment sequentially from the randomized list. If the parent/guardian(s) was interested in the study, an appointment was made for a screening interview at our study clinic. Enrollment occurred between November 2004 and April 2005. Study physicians recruited children if they fulfilled all of the following eligibility criteria: 1) age 1 to 10 years; 2) agreement to come to the study clinic for any febrile episode or illness; 3) agreement to avoid medications administered outside the study; 4) agreement to remain in Kampala during the study period; 5) no known side effects to the study medications; 6) weight ≥ 10 kg; 7) absence of severe malnutrition; 8) absence of known serious chronic disease; 9) absence of life threatening screening laboratory results; and 10) willingness of parent or guardian to provide written informed consent.

The study received ethical approval from the Uganda National Council of Science and Technology, the Makerere University Research and Ethics Committee, and the University of California, San Francisco Committee on Human Research.

### Follow-up of study participants, malaria treatment allocation and administration

Parents and guardians were asked to bring participants to the study clinic for all medical care. At each participant encounter a standardized system was used to assess participant symptoms, physical exam findings and laboratory abnormalities based on grading of severity (normal, mild, moderate, severe, life threatening). In general, events were classified as mild if no therapy was required, as moderate if minimal intervention and/or monitoring were required, as severe if medical care and possible hospitalization were required, and as life-threatening if active medical intervention or hospitalization were required. Weakness, anorexia, vomiting, diarrhoea, cough, pruritus, and coryza were actively assessed in all participants. Headache, abdominal pain, and nausea were also assessed in children over three years of age. A standardized physical exam was also conducted, which included assessment of hearing and fine finger dexterity (ability to pick up a small object). Any additional symptoms reported by the participant or exam findings were also assessed and graded accordingly.

If a participant presented with fever, a blood smear was taken, and malaria diagnosed if any of the following criteria were met: 1) complicated malaria (presence of severe malaria or danger signs) and any parasitaemia; or 2) fever (documented tympanic temperature ≥38.0°C and/or history of fever in the previous 24 hours) and any parasitaemia [25]. Following the diagnosis of their first episode of uncomplicated malaria, study participants were randomly assigned to receive one of three antimalarial regimens: amodiaquine plus sulphadoxine-pyrimethamine (AQ+SP), artesunate + amodiaquine (AS+AQ), or artemether-lumefantrine (AL).

Study medications were administered according to weight-based guidelines. The study medications were dosed as follows: amodiaquine (Camoquin; Parke-Davis, USA), 10 mg/kg on first two days then 5 mg/kg on third day, sulphadoxine-pyrimethamine (Fansidar; Hoffman-LaRoche, USA), sulphadoxine 25 mg/kg and pyrimethamine 1.25 mg/kg as a single dose on first day; artesunate (Arsumax; Sanofi-Aventis, France), 4 mg/kg on all three days; artemether-lumefantrine (Coartem; Novartis, Switzerland), 20/120 mg tablets given twice a day for three days according to weight: 5–14 kg, one tablet per dose; 15–24 kg, two tablets per dose; and 25–34 kg, three tablets per dose. Participants in the AQ+SP and AS+AQ groups also received placebo tablets administered in the evening over three days, dosed similarly to weight-based guidelines for AL. Administration of each first daily dose of medication was directly observed by the study nurses, and each second daily dose of medication or placebo was given to the participant's parent or guardian to administer at home in the evening. All study personnel involved in outcome assessment were blinded to treatment allocation and participants were not informed of their treatment assignments.

### Adverse event monitoring

Participants randomized to treatment with study medications were assessed for adverse events beginning with the first malaria episode. An adverse event was defined as any untoward medical occurrence, irrespective of its suspected relationship to the study medications as per International Conference of Harmonization (ICH) guidelines [27]. A serious adverse event was defined as any adverse experience that resulted in death, life threatening experience, participant hospitalization, persistent or significant disability or incapacity, or specific medical or surgical intervention to prevent serious outcome.

### Active surveillance for adverse events

On the day that treatment was initiated (day 0), a baseline assessment was conducted consisting of the standardized history and physical examination, and laboratory testing (complete blood count, measurement of alanine aminotransferase [ALT]). Participants were asked to return for completion of treatment and standardized follow-up assessment on days 1, 2, 3, 7, 14, or any other day they felt ill. Complete blood count and ALT measurement were repeated on day 14.

At each follow-up encounter, adverse events were identified by evaluating for any new or worsening symptoms, physical exam findings, or laboratory abnormalities, as compared to the day 0 baseline assessment. Participants with abnormalities present on day 0 were not classified as experiencing an adverse event unless the symptom worsened from baseline, or resolved and then recurred. For adverse events of moderate or greater severity, additional information was captured, including the suspected relationship of the event to the study treatment (unrelated, possible, probable, definite), event outcome, and date of resolution. Participants who experienced a serious adverse event were placed on study treatment hold until the relationship of the event to the study medications could be established. If the event was deemed unrelated or only possibly related to the study medications, participation in the study was continued. Patients with clinical treatment failure (according to 2006 WHO criteria) within 14 days of initiation of therapy were treated with quinine, and active surveillance for adverse events was continued for an additional 14 days [3].

### Passive surveillance for adverse events

Following the initial 14-day period, participants were asked to return to the clinic only when they desired medical attention. Passive surveillance for adverse events of moderate or greater severity continued until the participant was retreated with study medications, at which time a new cycle of adverse event assessment and reporting began, until the end of the study period, or until premature withdrawal from the study. Any case of symptomatic malaria diagnosed more than 14 days after a previous episode was considered a new malaria episode (for treatment purposes) and managed accordingly. Once randomized, participants received the same treatment regimen for all subsequent episodes of uncomplicated malaria.

### Laboratory testing

Complete blood counts and alanine aminotransferase measurements were performed by a College of American Pathologists accredited laboratory using a Coulter AcT 5 diff CP (Beckman Coulter) for haematology tests and Cobas Integra 400 plus (Roche) for chemistries.

### Statistical analysis

Details on sample size calculation are presented elsewhere [25]. Data were double-entered in Access (Microsoft Corporation, Redmond, Wash), and statistical analysis was performed using Stata version 8.0 (Stata Corporation, College Station, TX, USA). Data were evaluated with an intention-to-treat analysis including all participants with uncomplicated malaria who were randomized to treatment with study medications. Safety and tolerability outcomes include risk of adverse events at 14 days (active surveillance) and 42 days (passive surveillance) of follow-up. Specific adverse events were limited to the first occurrence of an adverse event after each treatment with study medications.

Data were analysed in a stepwise approach. All adverse events of any severity or relationship at 14 days of follow-up were first considered, then the analysis was restricted for adverse events of moderate or greater severity, and finally restricted for adverse events of possible, probable, or definite relationship to study medications. Risks of adverse events at 14 days were estimated using simple proportions as nearly all patients completed 14 day follow-up. Data at 42 days of follow-up was limited to adverse events of moderate or greater severity. Risks of adverse events at 42 days of follow-up were estimated using the Kaplan-Meier product limit formula, censoring for participants who did not complete 42 day follow-up either due to repeat treatment with study drugs or premature withdrawal from the study.

Pairwise comparisons of adverse events for individual episodes of malaria were made at 14 and 42 days of follow-up with generalized estimating equations, adjusting for repeated measures in the same study participant using exchangeable correlation and robust standard errors. A p-value of < 0.05 was considered statistically significant.

## Results

### Study population

**Figure 1:**
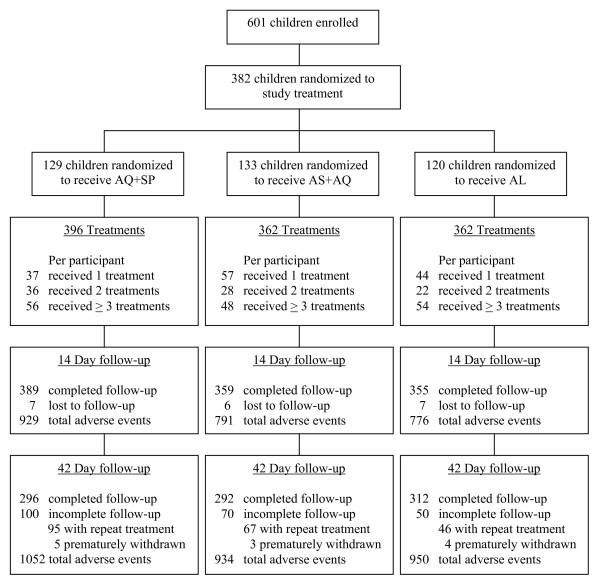
Trial Profile.

Details of recruitment and the study population are presented elsewhere [25,26]. Briefly, of 743 children screened, 142 were excluded from the study. The most common reasons for exclusion during screening included: parent/guardian unwilling to provide informed consent (30%), plans to move out of Kampala (20%), and weight < 10 kg (19%). A total of 601 children were enrolled from 323 households. Of these, 382 were diagnosed with at least one episode of uncomplicated malaria over the period of follow-up and were randomized to one of the three treatment groups (Figure 1).

### Study treatment follow-up

A total of 1120 study treatments were administered during 605 person-years of follow-up (Figure 1). The median number of treatments administered per participant treated at least once with study medications was 4 (range 1–20). The median age of participants receiving study treatments was 6.3 years (range 1.1 – 12.3 years), and was similar between the treatment arms. Nearly all (98%) participants completed 14 day follow-up for assessment of adverse events. Most participants (80%) completed 42 day follow-up; of the remaining participants, follow-up for 19% ended before 42 days when they were retreated with study medications, and 1% were prematurely withdrawn from the study. Reasons for premature withdrawal included movement out of the study area (8 participants), loss to follow-up (2), non-compliance (1), and withdrawal of consent (1).

### Risk of adverse events at 14 days

During the first 14 days after therapy, a total of 2496 adverse events, defined as any untoward medical occurrence, irrespective of suspected relationship to study medications, were detected. Most events were mild; only 749 (30%) were of moderate or greater severity. Of these events, 143 (19%) were classified as not related, 541 (72%) as possibly related, and 65 (9%) as probably related to study medications. No adverse events were considered to be definitely related to the study medications.

Statistically significant differences between the treatment groups were found for 4 adverse events: anorexia, subjective fever, weakness, and elevated temperature (Table 1). Within the first 14 days, participants treated with AQ+SP had a greater risk, compared to those treated with AL, of anorexia, weakness, and subjective fever, and a greater risk, compared to those treated with AS+AQ, of weakness and subjective fever. Participants treated with AL had a higher risk of elevated temperature than those receiving AS+AQ.

**Table 1:** Risk (%) of adverse events associated with study treatment within the first 14 days of treatment, including events of any severity grade

	**AQ+SP (n = 396)**	**AS+AQ (n = 362)**	**AL (n = 362)**
**Signs and symptoms**			

Coryza	115 (29.0%)	125 (34.5%)	118 (32.6%)
Cough	119 (30.1%)	108 (29.8%)	117 (32.3%)
Anorexia*	115 (29.0%)	89 (24.6%)	67 (18.5%)
Weakness^†^	93 (23.5%)	58 (16.0%)	47 (13.0%)
Abdominal pain	77 (19.4%)	56 (15.5%)	63 (17.4%)
Vomiting	64 (16.2%)	49 (13.5%)	41 (11.3%)
Subjective fever^‡^	59 (14.9%)	31 (8.6%)	33 (9.1%)
Headache	45 (11.3%)	38 (10.5%)	50 (13.8%)
Rash	38 (9.6%)	43 (11.9%)	46 (12.7%)
Generalized pruritus	34 (8.6%)	39 (10.8%)	30 (8.3%)
Diarrhoea	34 (8.6%)	29 (8.0%)	31 (8.6%)
Elevated temperature^γ^	20 (5.1%)	14 (3.9%)	31 (8.6%)
Nausea	12 (3.0%)	14 (3.9%)	13 (3.6%)
Hepatosplenomegaly	6 (1.5%)	9 (2.5%)	4 (1.1%)

Laboratory abnormalities**			

Neutropaenia	27/381 (7.1%)	18/345 (5.2%)	13/343 (3.8%)
Anaemia	9/393 (2.3%)	20/356 (5.6%)	11/357 (3.1%)
Elevated ALT	2/392 (0.5%)	1/358 (0.3%)	2/357 (0.6%)
Thrombocytopaenia	1/389 (0.3%)	0/350	0/351

**Serious adverse events**	7 (1.8%)	4 (1.1%)	4 (1.1%)

* Anorexia: AQ+SP vs. AL, p = 0.001

† Weakness: AQ+SP vs. AS+AQ, p = 0.015; AQ+SP vs. AL, p < 0.001

‡ Subjective Fever: AQ+SP vs. AS+AQ, p = 0.010; AQ+SP vs AL, p = 0.017

γ Elevated Temperature: AL vs. AS+AQ, p = 0.013

** Laboratory data not available on all participants

### Laboratory tests at 14 days

At 14 days, there were no statistically significant differences in the risk of laboratory-related adverse events between treatment groups (Table 1). However, noteworthy abnormalities in neutrophil counts were repeatedly observed in one subject. This participant, randomized to AS+AQ, experienced neutropaenia after six of 10 treatments; four episodes were of mild severity (750–1200 cells/mm^3^), one was moderate (660 cells/mm^3^), and one was severe (330 cells/mm^3^). For nine of the 10 treatments, the initial day 0 neutrophil count was within normal limits. All episodes of neutropaenia resolved spontaneously. The participant was subsequently excluded from the study due to concern about a causal association between AS+AQ and the neutropaenic episodes. Moderate grade neutropaenia was only seen in one other participant, after treatment with AQ+SP. This participant developed neutropaenic episodes after two of 15 study treatments, with one mild, and one moderate decrease in neutrophil count.

### Serious adverse events at 14 days

A total of 15 serious adverse events (SAEs) occurred within the first 14 days of follow-up (Table 1). Convulsions were most commonly reported, with eight episodes occurring in as many participants, all below seven years of age. The majority of the convulsions (75%) occurred in association with fever, and seven were classified as possibly related to study medications. One participant with a non-febrile convulsion was later found to have epilepsy, and in another case a complete history was not available. Two participants treated with AQ+SP developed significant weakness possibly related to study medications. Another two participants had bronchopneumonia requiring inpatient hospitalization. Additional serious events included severe vomiting, ALT elevation (thought to be an infectious hepatitis), and a traumatic bone fracture. No serious adverse event was considered to be probably or definitely related to study medications, and no deaths occurred.

### Risk of adverse events (of moderate or greater severity) at 14 days

To focus on clinically relevant adverse events occurring within the first 14 days of follow-up, the risk of developing events of moderate or greater severity was analysed (Table 2). Although the risks of more severe events were lower, the comparative differences between the treatment groups were similar. Nearly all of the previously described differences for risk of anorexia, weakness, subjective fever, and elevated temperature remained significant, and the associations strengthened. Further restricting the analysis, by focusing on events of moderate or greater severity which were possibly or probably related to study treatment, provided similar results.

**Table 2:** Risk (%) of adverse events (of moderate or greater severity) associated with study treatment within the first 14 days of treatment

	**AQ+SP (n = 396)**	**AS+AQ (n = 362)**	**AL (n = 362)**
**Signs and symptoms**			

Coryza	18 (4.6%)	29 (8.0%)	21 (5.8%)
Cough	30 (7.6%)	45 (12.4%)	37 (10.2%)
Anorexia*	39 (9.9%)	24 (6.6%)	15 (4.1%)
Weakness^†^	37 (9.3%)	20 (5.5%)	9 (2.5%)
Abdominal pain	11 (2.8%)	8 (2.2%)	6 (1.7%)
Vomiting	15 (3.8%)	5 (1.4%)	7 (1.9%)
Subjective fever^‡^	59 (14.9%)	31 (8.6%)	33 (9.1%)
Headache	2 (0.5%)	5 (1.38%)	4 (1.1%)
Rash	15 (3.8%)	10 (2.8%)	11 (3.0%)
Generalized pruritus	21 (5.3%)	19 (5.3%)	16 (4.4%)
Diarrhoea	0	4 (1.1%)	5 (1.4%)
Elevated temperature^γ^	14 (3.5%)	8 (2.2%)	23 (6.4%)
Nausea	4 (1.0%)	1 (0.3%)	1 (0.3%)
Hepatosplenomegaly	1 (0.3%)	1 (0.3%)	1 (0.3%)

Laboratory abnormalities**			

Neutropaenia	1/381(0.3%)	2/345 (0.6%)	0/343
Anaemia	4/393 (1.0%)	5/356 (1.4%)	1/357 (0.3%)
Elevated ALT	0/392	0/358	1/357 (0.3%)
Thrombocytopaenia	1/389 (0.3%)	0/350	0/351

* Anorexia: AQ+SP vs. AL, p = 0.004

† Weakness: AQ+SP vs. AL, p < 0.001

‡ Subjective Fever: AQ+SP vs. AS+AQ, p = 0.010; AQ+SP vs AL, p = 0.017

γ Elevated Temperature: AL vs. AS+AQ, p = 0.007

** Laboratory data not available on all participants

There were no statistically significant differences in laboratory abnormalities of moderate or greater severity between study treatments. There were three episodes of moderate or severe neutropaenia (described above), 10 episodes of moderate or severe anaemia, and one episode of severe thrombocytopaenia. One participant treated with AL developed severe ALT elevation at 14 days of follow-up, which was deemed possibly related to study medications, although an infectious hepatitis was suspected.

### Factors associated with adverse events

We assessed for factors which might modify the effect between treatment and risk of adverse events. Neither the number of prior treatments, nor duration since prior treatment, significantly impacted the risk of adverse events. However, when the analysis of the association between treatment and risk of adverse events was stratified by age, significant differences were found (Table 3). The association between treatment with AQ+SP and anorexia or weakness was primarily seen in younger children; while the association between AQ+SP treatment and subjective fever, and AL treatment and elevated temperature was observed in older children. For young children, the risk of anorexia associated with AQ+SP treatment was nearly four times higher than with AL, and two times higher than with AS+AQ. Similarly, the risk of weakness for young children treated with AQ+SP was over five times higher than for those treated with AL, and over two times higher than for AS+AQ-treated children.

**Table 3:** Association between study treatment and adverse events (of moderate or greater severity) within the first 14 days of treatment, stratified by age group

**Adverse events**	**Comparison groups**	**Age < 5 years**		**Age ≥ 5 years**	
		
		**RR (95% CI)**	**p-value**	**RR (95% CI)**	**p-value**
Anorexia	AQ+SP vs. AL	3.82 (1.59–9.17)	0.003	1.73 (0.79–3.81)	0.17
	AQ+SP vs. AS+AQ	2.10 (1.04–4.23)	0.04	1.32 (0.64–2.71)	0.46

Weakness	AQ+SP vs. AL	5.40 (1.86–15.7)	0.002	3.08 (1.17–8.14)	0.02
	AQ+SP vs. AS+AQ	2.26 (1.01–5.05)	0.04	1.68 (0.76–3.69)	0.20

Subjective fever	AQ+SP vs. AL	1.55 (0.78–3.10)	0.21	1.78 (1.08–2.93)	0.02
	AQ+SP vs. AS+AQ	1.22 (0.64–2.31)	0.54	2.80 (1.53–5.12)	0.001

Elevated temperature	AL vs. AQ+SP or AS+AQ	1.34 (0.60–3.03)	0.47	3.66 (1.59–8.41)	0.002

### Risk of adverse events at 42 days

Passive surveillance for adverse events of moderate or greater severity continued until participants were diagnosed with another episode of uncomplicated malaria, or study follow-up ended. During 15–42 days of follow-up after study medication treatment, an additional 440 adverse events were recorded, representing 15% of total adverse events captured. No life-threatening adverse events were observed. No events suspected to be probably or definitely related to study medications were detected. During 15–42 days of follow-up, an additional three SAEs were reported. Of these, one event was a febrile convulsion; another was elevation of liver enzymes secondary to acute hepatitis A infection, and the last a skin abscess.

At 42 days, patients treated with AQ+SP remained at significantly higher risk of anorexia and weakness than AL-treated participants; however, the difference in risk of subjective fever was not sustained (Table 4). AL treatment remained associated with a higher risk of elevated temperature as compared to treatment with AS+AQ and AQ+SP. In addition, a difference in the risk of diarrhoea emerged in the 42-day analysis, with a higher risk of diarrhoea associated with AL than AQ+SP treatment (p = 0.021).

**Table 4:** Risk (%) of adverse events (of moderate or greater severity) associated with study treatment within the first 42 days of treatment^Δ^

**Adverse events**	**AQ+SP (n = 396)**	**AS+AQ (n = 362)**	**AL (n = 362)**
Coryza	10.0%	12.2%	13.1%
Cough	14.7%	22.8%	20.9%
Anorexia*	10.3%	9.7%	5.5%
Weakness^†^	9.7%	6.5%	3.1%
Abdominal pain	6.0%	3.3%	3.4%
Vomiting	3.8%	1.7%	1.9%
Subjective fever	24.9%	22.8%	24.2%
Headache	1.5%	2.8%	3.4%
Rash	6.0%	5.5%	4.7%
Generalized pruritus	7.4%	8.2%	6.5%
Diarrhoea^‡^	0.7%	1.1%	3.2%
Elevated temperature^γ^	4.8%	4.5%	9.2%
Nausea	1.0%	0.3%	0.3%
Hepatosplenomegaly	0.3%	0.3%	0.3%

^**Δ**^Risk estimated by Kaplan-Meier product limit formula

* Anorexia: AQ+SP vs. AL, p = 0.005

† Weakness: AQ+SP vs. AL, p < 0.001

‡ Diarrhoea: AL vs. AQ+SP, p = 0.021

γ Elevated Temperature: AL vs. AQ+SP, p = 0.029; AL vs. AS+AQ, p = 0.008

## Discussion

In this randomized, blinded, longitudinal clinical trial, the safety and tolerability of three commonly used antimalarial combinations in Ugandan children was evaluated. Although all three regimens were found to be safe, participants treated with AQ+SP, particularly young children, were significantly more likely to develop anorexia and weakness than participants treated with AL or AS+AQ. Focusing the analysis on events of moderate or greater severity strengthened the observed associations, but redefining outcomes based on suspected relationship to study medications was of limited utility. Extending follow-up for adverse events from 14 to 42 days had little impact on the findings. Overall, in our cohort of children in Kampala, AL and AS+AQ appear to be superior to AQ+SP due to improved tolerability and greater efficacy [25].

ACTs are generally considered to be safe and well-tolerated, which is supported by this study [17,28,29]. The finding that participants treated with AL were more likely to have elevated temperature may be due to the known anti-pyretic properties of amodiaquine, which was a component of the other two study regimens. Persisting fever after treatment with AL could contribute to the perception that the combination is not as effective as other regimens, which may need to be addressed in training of health care workers and community sensitization. The higher risk of diarrhoea observed in AL-treated patients in the 42-day analysis has previously been reported, although it is unclear why the onset of this adverse event was delayed [30,31].

Although no significant differences in safety were found between AS+AQ and AL in the cohort, the one case of repeated neutropaenic episodes associated with AS+AQ was concerning given the known propensity of amodiaquine to cause blood dyscrasias and agranulocytosis [8]. A recent study in Uganda found a significantly increased risk of moderate and severe neutropaenia in HIV-infected children (also receiving co-trimoxazole prophylaxis and antiretroviral therapy) who were treated with AS+AQ, raising concern about use of AS+AQ for the treatment of malaria in HIV-infected individuals [32]. As newer ACTs are developed for the treatment of malaria, continued evaluation of the safety and tolerability of these combination therapies in clinical trials will be important.

In this study, a higher risk of anorexia and weakness associated with AQ+SP when compared with the other regimens was found, consistent with prior reports of severe weakness in Rwandan children treated with AQ+SP [20,28]. However, our findings suggest that the tolerability of AQ+SP may be age-dependent, with younger children significantly more likely to experience anorexia and weakness when treated with AQ+SP than with the ACT regimens. The finding that AQ+SP was associated with a higher risk of subjective fever in older children may reflect imprecision in obtaining a history of fever. Older children may have reported an overall syndrome of "feeling unwell", including subjective fever, while caregivers of younger children may have only reported the presence of subjective fever if the child felt hot. Although differences in the tolerability between AQ+SP and ACTs were found, no significant differences in the safety of these regimens were seen. In East Africa, where recent studies have also raised concerns about the effectiveness of AQ+SP, ACTs are now preferable [20,25,33]. However, in regions of Africa where both drugs remain effective and/or AQ+SP has been shown to have superior efficacy, the optimal choice for first-line treatment will need to be made considering all relevant factors, including efficacy, safety and tolerability, cost, and availability [6,34].

No standardized system for adverse event monitoring in antimalarial clinical trials currently exists, and the approach to monitoring may differ between sites. Guidelines for use of laboratory testing in antimalarial drug safety monitoring are also lacking.  In this study, events were actively assessed at all follow-up visits, with interviews, a standard physical exam, and laboratory tests at standard intervals. Although time intensive, the system allowed events related to drug tolerability, such as anorexia and weakness, to be captured. Relying on spontaneous reporting of symptoms by participants, or assessing only for objective measures, such as serious adverse events and laboratory abnormalities, may have missed differences in tolerability. The duration of follow-up for monitoring of adverse events after antimalarial therapy is variable, often coinciding with the recommended length of follow-up for efficacy outcomes of 28 or 42 days [17,19,20,28]. Few differences in the comparative risk of adverse events at 14 and 42 days of follow-up were found. All study medications were safe at 14 days of follow-up, and, except for the emergence of a risk of diarrhoea in participants treated with AL, extending the follow-up period to 42 days did not identify any changes in safety, or tolerability compared to 14-day outcomes.

There were several potential limitations to this study. Although the assessment of adverse events was designed to be as objective as possible by using grading scales for severity and relationship, the results are limited by the subjective interpretations of symptom reports. In addition, active surveillance continued only for the 14 days after study treatment was initiated. Extending the duration of active surveillance may have captured additional events of interest. Laboratory values were only assessed at day 0 and day 14 of study treatment, routinely every 90 days, and at the discretion of the study clinicians. Some abnormalities may not have been captured by this schedule. Although 382 patients treated with study medications over multiple treatments were followed, the relatively small sample size limited the ability to detect uncommon events. Finally, multiple comparisons in the risk of adverse events associated with treatment groups were made, increasing the likelihood that statistically significant differences in risks could have been detected just by chance.

## Conclusion

This study confirms the safety and tolerability of AS+AQ and AL in Ugandan children, and suggests that AQ+SP is safe, but less well-tolerated than the other regimens, particularly in young children. The higher risk of elevated temperature observed in association with AL could impact on the perception of its effectiveness and may need to be addressed. Although no significant differences in safety were found between AS+AQ and AL, the one case of repeated neutropaenic episodes associated with AS+AQ was concerning. As newer ACTs are deployed, evaluating for safety and comparative tolerability will be essential. Standardized guidelines for monitoring adverse events in antimalarial clinical trials are needed.

## Authors' contributions

PJR, GD, MRK, and SGS conceived and designed the study, CMS, TDC, DNM, BN, and SGS participated in data collection, GD, TDC, PJ, and VY participated in the data analysis, CMS, PJ, TDC, AOT, PJR, GD, and SGS participated in interpretation of the data. All authors participated in the writing of the manuscript, and read and approved the final manuscript. None of the authors declare any potential conflicts of interest, including financial interests and relationships and affiliations relevant to the subject of the manuscript.
